# Factors Associated With Cervical Cancer Screening in Puerto Rico

**Published:** 2010-04-15

**Authors:** Ana P. Ortiz, Sarah Hebl, Ruby Serrano, María E. Fernandez, Erick Suárez, Guillermo Tortolero-Luna

**Affiliations:** University of Puerto Rico Comprehensive Cancer Center and University of Puerto Rico Medical Sciences Campus; Tufts University School of Medicine, Boston, Massachusetts; Puerto Rico Behavioral Risk Factor Surveillance System, Puerto Rico Department of Health, San Juan, Puerto Rico; University of Texas at Houston, Houston, Texas; University of Puerto Rico Medical Sciences Campus, San Juan, Puerto Rico; University of Puerto Rico Comprehensive Cancer Center, San Juan, Puerto Rico

## Abstract

**Introduction:**

Racial/ethnic disparities in cervical cancer screening exist in the United States; rates are lowest among women who live in Puerto Rico. We identified factors associated with cervical cancer screening among women aged 18 years or older living in Puerto Rico.

**Methods:**

We included women who participated in the Puerto Rico Behavioral Risk Factor Surveillance System in 2006 who had not had a hysterectomy (n = 2,206). We calculated the weighted population prevalence estimates of Papanicolaou (Pap) test screening in the past 3 years and used logistic regression models to assess factors associated with screening.

**Results:**

Most participants (71.9% [95% confidence interval (CI)Most participants (71.9% [95% confidence interval (CI) = 69.4%-74.4%]) reported having had a Pap test in the preceding 3 years. Factors associated with screening in multivariate analysis included routine checkup in the past year and leisure-time physical activity. Compared with women with a household income less than $15,000, those with higher incomes were more likely to have had a Pap test. Similarly, divorced or separated women were more likely to have been screened (odds ratio [OR] = 1.13; 95% CI = 1.12-1.15) than those who were married/living together. We did not find associations between screening behavior and education, health care coverage, body mass index, or smoking status.

**Conclusion:**

The prevalence of cervical cancer screening in Puerto Rico is below the 90% recommendation established by *Healthy People 2010*. Our findings regarding factors associated with Pap screening behavior identified population subgroups who are underscreened and who may benefit from targeted interventions and screening programs.

## Introduction

In the United States, the incidence of cervical cancer among Hispanics (14.2 per 100,000) is almost double that of non-Hispanic whites (7.3 per 100,000); the death rate for Hispanics (3.4 per 100,000) is also 50% higher than for non-Hispanic whites (2.3 per 100,000) (1). Despite the overall decline in cervical cancer incidence and deaths in the United States in the last few decades, Hispanic women are less likely to be diagnosed with localized disease and have poorer survival rates than non-Hispanic whites (1,2). Cervical cancer is the 6th most common cancer among Hispanic women and ranks only 13th among cancers for non-Hispanic whites (2). In Puerto Rico, cervical cancer is the fourth most commonly diagnosed cancer in women, accounting for 4% of all newly diagnosed cancers and 2% of all cancer-related deaths among women (3).

The use of the Papanicolaou (Pap) test has resulted in a substantial decline in cervical cancer illnesses and deaths over recent decades (2). In the United States, lack of cervical cancer screening is the most powerful predictor of cervical cancer; thus, disparities in Pap test coverage influence disparities in cervical cancer (4). Despite the accessibility of this screening method, racial/ethnic disparities exist in its use in the United States (5). For example, in 2004, women living in Puerto Rico had the lowest prevalence of having had a recent (last 3 years) Pap test (73%) when compared with non-Hispanic whites (87%), African Americans (89%), and Hispanics (87%) living in the United States (excluding US territories) (6). Among Hispanics and other racial/ethnic groups in the United States, sociodemographic factors such as older age, lower income, lower education level (particularly those who did not graduate from high school), and lack of health care coverage have been positively correlated with a lower rate of Pap screening (7,8). In addition to sociodemographic factors, psychosocial factors such as embarrassment, language barriers, fear, lack of knowledge, and perceived partner disapproval influence Hispanic women's use of Pap screening (9).

To the best of our knowledge, no study has explored the factors influencing Pap test use among women in Puerto Rico. The purpose of this study was to determine the factors associated with self-reported recent Pap test (within 3 years before the interview) among women aged 18 years or older living in Puerto Rico who participated in the Puerto Rico Behavioral Risk Factor Surveillance System (PR-BRFSS). The results of our study are needed to elucidate barriers to cervical cancer screening in the Hispanic population of Puerto Rico. Our objectives are in alignment with the National Breast and Cervical Cancer Early Detection Program's goal of reducing racial disparities in screening and early detection and with the *Healthy People* *2010* goal of reducing health disparities (10). In addition, this study contributes to meeting the objective of the Puerto Rico Comprehensive Cancer Control Plan (11) of increasing the proportion of women aged 18 years or older who receive a Pap test consistent with current recommendations.

## Methods

For this study, we used 2006 data from the PR-BRFSS (12). The PR-BRFSS is part of the national BRFSS, a state-based system of health surveys established in 1984 by the Centers for Disease Control and Prevention to collect information on health risk behaviors, preventive health practices, and health care access primarily related to chronic disease and injury. This cross-sectional survey is conducted annually among noninstitutionalized adults, aged 18 years or older, in all 50 states, the District of Columbia, Puerto Rico, the US Virgin Islands, and Guam. The institutional review board of the University of Puerto Rico Medical Sciences Campus approved this study.

The women eligible for this study had participated in the 2006 PR-BRFSS, were aged 18 years or older, had no history of hysterectomy, and had responded to the question of whether or not they had had a Pap test in the past 3 years before the interview. Among the 3,040 women aged 18 years or older who participated in the PR-BRFSS in 2006, we excluded 710 women who had had a hysterectomy and 124 women with missing information regarding age (n = 16), Pap test history (n = 38), time since last Pap test (n = 31), or hysterectomy status (n = 39), which left a final study sample of 2,206 women.

The outcome variable of interest for this analysis was the proportion of women who had had a Pap test in the 3 years before the interview. Factors of interest included demographic characteristics, such as age in years (18-20, 21-30, 31-40, 41-50, 51-60, ≥61), marital status (married/living together, divorced/separated, widowed, single), educational attainment (less than high school graduate, high school graduate/General Educational Development certification, some college/technical school, college graduate), household income (<$15,000, $15,000-$34,999, $35,000-$49,999, ≥$50,000), health care coverage (yes/no), employment status (currently employed, unemployed, homemaker or retired, unable to work, student), and number of children living in the household (0, 1, 2, ≥3). Clinical characteristics included body mass index (BMI, categorized as normal or underweight [18.5-24.9 kg/m^2^], overweight [25.0-29.9 kg/m^2^], or obese [≥30.0 kg/m^2^]), routine checkup in the past year (yes/no), and perceived general health status (fair or poor, good or excellent). Lifestyle characteristics included current smoking status (yes/no), binge drinking (4 or more drinks on 1 occasion [yes/no]), and leisure-time physical activity in the past 30 days (yes/no).

We conducted the statistical analysis by using SAS version 9.2 (SAS Institute, Inc, Cary, North Carolina) and Stata version 10.0 (StataCorp LP, College Station, Texas). We first described the study sample according to demographic, clinical, and lifestyle characteristics by using the survey frequency function in SAS. We then assessed the relationship between cervical cancer screening behavior and the demographic, clinical, and lifestyle factors by using contingency tables and Pearson's χ^2^ tests, which also required the use of the survey frequency function. To further assess these relationships, we used the generalized linear model procedure in Stata to construct simple and multivariable logistic regression models (13). We estimated the prevalence odds ratios and their 95% confidence intervals to determine the magnitude of the association between the specific factors and cervical cancer screening behavior. The variables significantly associated with cervical cancer screening (*P* < .05) in the age-adjusted logistic regression models were included in the multivariable logistic regression models; those with at least marginal significance (*P* < .10) in the multivariate model were retained in the model. All data were weighted according to the respondent's age and the inverse of her probability of selection by using the 2006 census population projections. Detailed information about BRFSS weighting procedures can be found in the BRFSS operational guide (14).

## Results

Approximately half of the women were aged 40 years or younger and were married or living as a couple  (Table 1). Most had a household income of less than $35,000 a year, and almost all reported having health care coverage. Two-thirds of women reported that they were in good to excellent general health; 81% had a routine checkup in the previous year, 82% had at least 1 Pap test in their lifetime, and 72% had a Pap test in the past 3 years.

In the bivariate analysis, Pap screening in the past 3 years was significantly associated (*P* < .05) with age, marital status, education level, household income, employment status, number of children in the household, BMI, routine physical examination in the past year, health status, and leisure-time physical activity. Lower rates of Pap screening were observed in younger women, single women, women with a household income less than $15,000, women with some college or technical school education, and students (Table 2). In addition, lower rates of Pap test screening were reported by underweight or normal-weight women, women who had not had a routine medical checkup in the prior year, and those who reported no leisure-time physical activity. Pap screening was not associated with smoking status, health care coverage, binge drinking, or heavy alcohol consumption.

We found no significant interactions in the multivariate model (likelihood ratio χ^2^ = 70.13, *P* = .17). Compared with younger women (aged 18-20 years), older women (aged ≥21 years) were 2 to 5 times as likely to have had a Pap test in the last 3 years (Table 3). In addition, compared with women with the lowest household income, women with larger household incomes were also more likely to have had a Pap test in the past 3 years. Single women (never married) and widows were less likely than married women to have had a Pap test in the past 3 years.

## Discussion

In 2006, Puerto Rico fell short of meeting the *Healthy People 2010* goal of a 90% Pap test screening rate for women aged 18 years or older (15). Our study shows that younger (aged ≤30 years) and older (aged ≥61 years) Puerto Rican women reported lower rates of cervical cancer screening than women aged 31 to 60 years. These results are consistent with the overall screening patterns in the United States (16) and among Hispanic women in the rest of the United States (8). Of interest is the low prevalence of screening among women aged 18 to 20 years in Puerto Rico observed in our study (9%). This prevalence is consistent with the low rate of cervical cancer screening among women aged 18 to 24 years in Puerto Rico (41%) reported by the BRFSS for 2004, which in addition is much lower than the screening rate of their counterparts in the rest of the United States (median = 81%), a pattern that has been consistent since 1996 (6). In addition, women with a household income of at least $35,000 were 2 to 3 times as likely to have been screened as those with the lowest household income. Household income has been positively correlated with cervical cancer screening in multiple studies (10,16-17), an association that could be partially explained by improved access to care with increasing wealth.

In contrast to results in other populations, our study found no substantial positive effect of education, employment status, or health care coverage on screening practices (7,8,16,17). Although the reasons for the lack of association between screening behavior and education and employment are unknown, for health care coverage the lack of association may be explained by the high prevalence of women in our study who had health care coverage (94%). The health care reform legislation passed in Puerto Rico in 1993 made health insurance available for underserved populations in Puerto Rico at or below 200% of the poverty level.

Routine checkup in the past year increased the likelihood of having been screened, a result consistent with other studies (8,18). This finding is not surprising since cervical cancer screening is often recommended by the doctor during the clinical visit. Participating in leisure-time physical activity increased the likelihood of screening in our study by more than 60%, a result consistent with that of another recent study that found that exercise was positively associated with breast and cervical cancer screening (7). This previous study also found a positive association between nonsmoking and screening. Our research, however, did not observe this association. Although studies have consistently shown an inverse relationship between obesity and screening behavior (decreased cervical cancer screening with increasing body size) (19), we found no association between BMI and cervical cancer screening in multivariate analysis. While in some groups higher weight may be associated with less emphasis on health and thus less screening, our findings may reflect a cultural norm of acceptance of a larger body weight/body size in Puerto Rican women similar to that found is some populations of black women in the United States (20).

Our study is subject to limitations. First, because the BRFSS is a telephone-based survey, it includes data only from residents who have a working home telephone and, thus, is unable to survey those who reside in households without telephone access. Consequently, the above data may not be generalizable to the entire adult Puerto Rican female population. Evidence suggests that income is positively associated with a recent Pap test. Since women who do not have telephones are more likely to have lower incomes, they likely also have lower Pap screening rates than women who participated in the study (8). Nonetheless, response rates in the 2006 PR-BRFSS were much higher (74%) than the median reported for BRFSS surveys in all other US states and territories of the United States for the same year (51%) (21). Finally, the prevalence estimates were based on self-reported information, which is subject to recall bias and social desirability bias.

Our study shows that the rates of cervical cancer screening in Puerto Rico are lower than those in the United States, and well below the 90% goal established by *Healthy People* *2010*. We have identified factors associated with Pap test screening in Puerto Rico that help identify subgroups of the population who are underscreened and who would benefit from targeted interventions. Interventions should focus on increasing screening rates, particularly among young and low-income women. Future studies should also focus on other psychosocial correlates of screening, including attitudes, beliefs, and cultural norms regarding screening practices. Studies of the effect of social determinants of health, such as area of residence (rural vs urban) and migrant status, are warranted in this population, since these factors influence screening behavior (17,22). In addition, public health policy regarding universal mandatory cervical cancer screening coverage in Puerto Rico should be implemented.

## Figures and Tables

**Table 1:** Characteristics of 2,206 Adult Women With Data on Pap Testing, Puerto Rico Behavioral Risk Factor Surveillance System, 2006^a^

Characteristic	n^b^	% (95% CI)^c^
**Age, y**		
18-20	75	9 (6.6-10.5)
21-30	209	18 (15.3-20.0)
31-40	400	22 (20.1-24.3)
41-50	446	19 (17.2-20.8)
51-60	553	19 (17.4-20.8)
≥61	523	14 (12.2-14.8)
**Marital status**		
Married/living together	1,045	52 (49.0-54.1)
Divorced/separated	493	16 (14.7-17.9)
Widowed	343	9 (7.6-9.7)
Single	323	24 (20.9-26.0)
**Education**		
Less than high school graduate	632	22 (19.8-23.4)
High school graduate/GED	501	23 (21.0-25.3)
Some college/technical school	404	22 (19.6-24.0)
College graduate	668	33 (31.0-35.8)
**Household income**		
<$15,000	909	40 (37.3-42.4)
$15,000-34,999	683	41 (38.6-44.0)
$35,000-49,999	145	9 (7.5-10.7)
≥$50,000	150	10 (8.1-11.4)
**Health care coverage**		
Yes	2,093	94 (93.1-95.5)
No	110	6 (4.5-6.9)
**Employment status**		
Currently employed	803	41 (38.8-43.8)
Unemployed	103	5 (3.9-6.2)
Homemaker/retired	1,043	37 (34.8-39.4)
Unable to work	140	5 (3.9-5.6)
Student	116	12 (9.7-14.0)
**No. of children in household**		
0	1,327	54 (51.2-56.3)
1	354	20 (17.5-27.8)
2	345	17 (15.4-19.0)
≥3	180	9 (7.9-10.9)
**BMI**		
Underweight or normal weight (18.5-24.9 kg/m^2^)	805	45 (42.0-47.3)
Overweight (25.0-29.9 kg/m^2^)	695	32 (29.8-34.6)
Obese (≥30 kg/m^2^)	525	23 (21.0-25.2)
**Routine checkup in the past year**		
Yes	1,755	81 (78.8-83.0)
No	372	19 (17.0-21.2)
**General health status**		
Good to excellent	1,298	67 (64.8-69.2)
Fair to poor	902	33 (30.8-35.2)
**Ever had a Pap test**		
Yes	1,957	82 (79.7-84.4)
No	249	18 (15.6-20.3)
**Had Pap test in past 3 years**		
Yes	1,695	72 (69.4-74.4)
No	511	28 (25.6-30.6)
**Binge drinking**		
Yes	117	7 (5.6-8.6)
No	2,042	93 (91.4-94.4)
**Heavy alcohol consumption**		
Yes	2,131	98 (96.8-98.5)
No	44	2 (1.5-3.2)
**Current smoking status**		
Yes	204	9 (7.8-10.7)
No	1,999	91 (89.3-92.2)
**Leisure-time physical activity**		
Yes	1,173	54 (51.0-56.0)
No	1,032	47 (44.0-49.0)

Abbreviations: Pap, Papanicolaou; CI, confidence interval; GED, General Educational Development certificate; BMI, body mass index.

a Inclusion criteria included no prior hysterectomy and a yes or no response to the BRFSS question regarding having had a Pap test in the past 3 years. All other participants were excluded.

b Totals may vary as a result of missing responses, including don't know/not sure and refused.

c Weighted population estimates and percentages.

**Table 2:** Percentage of Adult Women Who Have Had a Pap Test in the Past 3 Years, Puerto Rico Behavioral Risk Factor Surveillance System, 2006^a^

Characteristic	n^b^	% (95% CI)^c^
**Age (*P* < .001), y **		
18-20	25	26 (16.0-35.6)
21-30	147	64 (56.4-71.3)
31-40	322	81 (76.0-84.9)
41-50	366	81 (77.1-85.2)
51-60	461	83 (79.4-86.4)
≥61	374	69 (64.2-73.3)
**Marital status (*P* < .001)**		
Married/living together	874	83 (80.6-85.9)
Divorced/separated	406	82 (77.3-85.9)
Widowed	245	68 (62.2-74.0)
Single	168	41 (35.0-47.7)
**Education (*P* = .006)**		
Less than high school graduate	457	71 (66.7-75.0)
High school graduate/GED	382	69 (62.9-74.2)
Some college/technical school	306	67 (61.1-73.1)
College graduate	549	78 (73.8-82.0)
**Household income (*P* < .001)**		
<$15,000	672	70 (66.2-73.7)
$15,000-34,999	546	73 (68.9-77.6)
$35,000-49,999	134	87 (79.2-95.3)
≥$50,000	128	84 (77.1-91.6)
**Health care coverage (*P* = .25)**		
Yes	1,621	72 (69.7-74.9)
No	72	66 (56.1-76.6)
**Employment status (*P* < .001)**		
Currently employed	661	79 (75.9-82.9)
Unemployed	85	76 (65.2-87.7)
Homemaker/retired	799	77 (73.6-79.6)
Unable to work	110	80 (72.4-87.0)
Student	39	26 (17.6-33.8)
**Number of children in household (*P* = .002)**		
0	999	68 (64.3-71.3)
1	279	75 (69.6-80.7)
2	280	80 (75.3-84.9)
≥3	137	73 (65.1-81.2)
**BMI (*P* < .001)**		
Under or normal weight (18.5-24.9 kg/m^2^)	608	68 (63.5-72.1)
Overweight (25.0-29.9 kg/m^2^)	574	80 (75.9-83.6)
Obese (≥30 kg/m^2^)	403	74 (69.2-78.7)
**Routine checkup in the past year (*P* < .001)**		
Yes	1,409	76 (73.0-78.5)
No	241	60 (53.6-65.9)
**General health status (*P* = .045)**		
Good to excellent	999	70 (67.0-73.6)
Fair to poor	692	75 (71.8-78.6)
**Binge drinking (*P* = .29)**		
Yes	87	66 (55.5-77.2)
No	1,572	72 (69.5-74.7)
**Heavy alcohol consumption (*P* = .95)**		
Yes	34	72 (55.8-88.9)
No	1,639	72 (69.4-74.4)
**Current smoking status (*P* = .93)**		
Yes	204	72 (69.3-74.6)
No	1,999	72 (64.9-79.6)
**Leisure-time physical activity (*P* = .005)**		
Yes	945	75 (72.0-78.5)
No	750	68 (64.3-71.8)

Abbreviations: Pap, Papanicolaou; CI, confidence interval; GED, General Educational Development certificate; BMI, body mass index.

a Data exclude women who had had a hysterectomy.

b Totals may vary as a result of missing responses, including don't know/not sure and refused.

c Weighted population estimates; women who responded don't know/not sure, or who refused were excluded.

**Table 3:** Multivariate Predictors of Having Had a Pap Test in the Past 3 Years Among Adult Women, Puerto Rico Behavioral Risk Factor Surveillance System, 2006^a^

Predictor Variable	Multivariate OR (95% CI)
**Age group (*P* < .001), y**	
18-20	1 [Reference]
21-30	3.54 (3.46-3.62)
31-40	4.61 (4.50-4.72)
41-50	3.88 (3.79-3.98)
51-60	4.26 (4.15-4.37)
≥61	2.38 (2.31-2.44)
**Household income (*P* < .001)**	
<$15,000	1 [Reference]
$15,000-34,999	1.29 (1.28-1.31)
$35,000-49,999	2.78 (2.71-2.84)
≥$50,000	2.45 (2.39-2.50)
**Marital status (*P* < .001)**	
Married/living together	1 [Reference]
Divorced/separated	1.14 (1.12-1.15)
Widowed	0.64 (0.62-0.65)
Single	0.19 (0.18-0.19)
**Routine checkup in the past year (*P* < .001)**	
Yes	2.52 (2.49-2.55)
No	1 [Reference]
**Leisure-time physical activity (*P* < .001)**	
Yes	1.41 (1.40-1.42)
No	1 [Reference]

Abbreviations: Pap, Papanicolaou; OR, odds ratio; CI, confidence interval.

a Data exclude women who had had a hysterectomy and those who responded don't know/not sure or who refused to answer.
