# Erratum, Vol. 7: Issue 3

**Published:** 2010-08-15

In the article "Factors Associated With Cervical Cancer Screening in Puerto Rico," the categories of the age groups used for analysis were reported incorrectly. The age groups used for analysis were 18-20, 21-29, 30-39, 40-49, 50-64, and 65 or older instead of 18-20, 21-30, 31-40, 41-50, 51-60, and 61 or older. The authors regret this error. Corrections to the text and tables were made to our Web site on August 15, 2010, and appear online at www.cdc.gov/pcd/issues/2010/may/09_0123.htm.

En el artículo "Factores asociados a la detección del cáncer de cuello uterino en Puerto Rico" se notificaron incorrectamente las categorías de los grupos de edades usadas para el análisis. Los grupos de edades usados para el análisis fueron 18-20, 21-29, 30-39, 40-49, 50-64 y 65 o más, en vez de 18-20, 21-30, 31-40, 41-50, 51-60 y 61 o más. Los autores lamentan este error. Las correcciones en el texto y en las tablas se hicieron en nuestro sitio web en agosto 15, 2010 y se encuentran en Internet en www.cdc.gov/pcd/issues/2010/may/09_0123_es.htm.

